# Spatiotemporal patterns and factors contributing to neonatal mortality in Ethiopia: Data from EDHS 2000 to 2019

**DOI:** 10.1371/journal.pone.0310276

**Published:** 2024-11-04

**Authors:** Getiye Dejenu Kibret, Habtamu Mellie Bizuayehu, Abel F. Dadi, Erkihun Amsalu, Addisu Alehegn Alemu, Tahir Ahmed Hassen, Cheru Tesema Leshargie, Meless Gebrie Bore, Zemenu Yohannes Kassa, Daniel Bekele Ketema, Jemal E. Shifa, Animut Alebel, Kedir Y. Ahmed

**Affiliations:** 1 School of Public Health, College of Medicine and Health Sciences, Debre Markos University, Debre Markos, Ethiopia; 2 Australian Institute of Health Innovation, Faculty of Medicine, Health and Human Sciences, Macquarie University, Australia; 3 First Nations Cancer and Wellbeing Research (FNCWR) Research Program, School of Public Health, The University of Queensland, Australia; 4 Menzies School of Health Research, Charles Darwin University, NT, Australia; 5 Addis Continental Institute of Public Health, Addis Ababa, Ethiopia; 6 Sydney Medical School, Faculty of Medicine and Health, University of Sydney, Australia; 7 St. Paul Hospital Millennium Medical College, Addis Ababa, Ethiopia; 8 School of Women’s and Children’s Health, University of New South Wales, Sydney, Australia; 9 Center for Women’s Health Research, College of Health, Medicine and Wellbeing, The University of Newcastle, NSW, Australia; 10 School of Public Health, Faculty of Health, University of Technology Sydney, Ultimo, NSW, Australia; 11 School of Nursing and Midwifery, Faculty of Health, University of Technology Sydney, Ultimo, NSW, Australia; 12 School of Nursing, College of Medicine and Health Science, Hawassa University, Hawassa, Ethiopia; 13 The George Institute for Global Health, University of New South Wales (UNSW), Sydney, Australia; 14 Australasian Health Outcomes Consortium, Faculty of Science, Medicine and Health, University of Wollongong, Wollongong, NSW, Australia; 15 Rural Health Research Institute, Charles Sturt University, Orange, NSW, Australia; Bahir Dar University, ETHIOPIA

## Abstract

**Background:**

Although Ethiopia has substantial improvements in various health indicators such as maternal and child mortality, the burden of neonatal mortality remains high. Between 2016 and 2019, neonatal mortality increased from 29 deaths per 1,000 live births to 33 deaths per 1,000 live births. This study aimed to explore the spatial patterns and factors contributing to neonatal mortality in Ethiopia.

**Methods:**

Data from the Ethiopian Demographic and Health Surveys (EDHS) for the years 2000, 2005, 2011, 2016, and 2019 were analyzed. The EDHS sampling design uses a two-stage cluster sampling technique, considering census enumeration areas as primary sampling units and households as secondary sampling units. We used the Spatial Scan analysis in SaTScan and Getis-Ord Gi* statistic in Geographic Information System (GIS), to analyse the spatiotemporal patterns of neonatal mortality. Maternal, newborn and health service-related factors contributing to neonatal mortality were also analyzed using a multilevel logistic regression model. Adjusted Odds Rios (AOR) with corresponding 95% CI were presented as a measure of association and a P-value of 0.05 was used to declare statistical significance.

**Results:**

During the initial three consecutive surveys, there was a consistent pattern of hot spot clusters in the Amhara and Benshangul Gumuz regions, along with certain parts of the Oromia region. However, in later surveys, these clusters shifted to the eastern parts of the country, notably including the Somali region. Early initiation of breast feeding was associated with reduced chances of neonatal death (Adjusted Odds Ratio [AOR]) = 0.27; 95% Confidence Interval [CI]: 0.23, 0.32). Neonates born at home (AOR = 1.46; 95% CI: 1.16, 1.82) and male babies had a higher likelihood of mortality during the neonatal period compared to their counterparts (AOR = 1.36; 95% CI: 1.24, 1.51). The odds of neonatal mortality increased with the number of children a mother had ever given birth to (AOR = 1.36; 95% CI: 1.24, 1.51). In contrast, longer birth intervals were associated with a reduced risk of neonatal mortality (AOR = 0.76; 95% CI: 0.68, 0.83).

**Conclusion:**

The central southern, central-western, north-western, and northern parts of Ethiopia had most of the neonatal death clusters in the first three rounds of DHS while eastern Ethiopia had the highest neonatal mortality clusters in the latest two surveys. Our results underscore the importance for policymakers and health administrators to reassess intervention approaches and reallocate resources to regions identified as hot spots for neonatal mortality. Enhancing the initiation of breastfeeding within the first hour of birth would improve newborn survival rates. Special attention and care need to be given to babies born of smaller sizes.

## Introduction

Neonatal mortality, defined as the death of a newborn within the first 28 days of life, remains a critical global health concern [1]. The neonatal phase marks a critical period for the survival of newborns as they undergo a series of significant physiological transitions from the uterine environment to the outside world [2, 3]. The initial seven days following birth are particularly precarious and require utmost attention for neonatal survival [2, 4].

The Sustainable Development Goal-3 targets envisioned that by 2030, all countries worldwide will strive to eliminate preventable newborn deaths, significantly reducing neonatal mortality to a target rate of 12 deaths per 1,000 live births [5]. In line with this target, Ethiopia has set specific target of reducing neonatal mortality to a rate of 10 deaths per 1,000 live births between the period 2015/16 to 2019/20 [6]. However, despite substantial progress in various health indicators, the burden of neonatal mortality in Ethiopia continues to pose significant challenges [7, 8]. Instead of a decline, an increasing trend has been observed, with neonatal mortality rates rising from 29 deaths per 1,000 live births in 2016 to 33 deaths per 1,000 live births in 2019.

Variations in neonatal mortality across socio-economic and between different regions of the country are significant [7]. A range of factors also contributed to neonatal mortality, including socio-economic, socio-demographic and health services use-related factors [9–15]. This warrants the importance of continuous measurement to understand the drivers for this change and identifying areas with special priorities using spatial analysis techniques.

Understanding the geographic and temporal distribution of neonatal mortality would help targeting interventions and allocating resources effectively [16]. By identifying areas with higher rates and clusters of neonatal deaths as well as factors contributing to neonatal mortality, policymakers and healthcare providers can prioritise interventions and improve healthcare delivery to reduce neonatal mortality. Mapping temporal trends could help identify areas with persistently high neonatal mortality rates as well as those evolving, thereby enabling focused intervention strategies. The current study aims to explore the spatial and temporal patterns and identify factors associated with neonatal mortality. The findings would inform policymakers and local administrators to help better service administration and focused interventions.

## Methods

### Study setting, data source and population

Ethiopia is a low-income country with a population of more than 112 million as of the 2019 projection [17]. The study utilizes data from multiple EDHS survey rounds from the years 2000, 2005, 2011, 2016 and 2019 [18–22]. The EDHS provides comprehensive and nationally representative data on various health indicators, including neonatal mortality rates, collected through household surveys. The EDHS data sets were accessed through the Monitoring and Evaluation to Assess and Use Results, Demographic and Health Surveys (MEASURE DHS) project with permission [23]. The datasets for the neonatal mortality variable and the Global Positioning System (GPS) coordinate joined using the cluster level common identifier. The study population for each round of the survey were newborns in Ethiopia from birth to the 28^th^ day of birth and the primary outcome of interest is neonatal mortality.

### Outcome measures

The primary endpoint of this study was the rate of neonatal mortality per 1000 live births within the initial 28 days postpartum. The outcome variable is defined as a binary indicator of neonatal death occurring within 28 days of birth, categorised as ’yes’ for deaths and ’no’ for survivals. The secondary endpoint examined the geographical distribution of these neonatal deaths. The analysis incorporated potential contributors to neonatal mortality such as socio-economic attributes and health service-related factors (Table 1).

**Table 1:** Descriptions of study variables for factors associated with neonatal mortality in Ethiopia, EDHS 2000–2019.

Variable	Value levels	Descriptions
Neonatal survival status	0. Survived 1. Dead	Outcome variable defined either death within 28 days of birth or survived beyond
Place of residence	1. Urban 2. Rural	
Living situation of the mother	0. Living with a partner 1. Not living with a partner	Women who are divorced/separated/widowed are considered not living with a partner
Educational status of the mother	0. No education 1. Primary education 2. Secondary and above	
Age at first birth	Continuous	Age of the mother at first birth
Total children ever born	Continuous	The total number of children the mother ever had
Birth order/rank	Continuous	The order of the newborn relative to their siblings
Birth type	0. Singleton 1. Twin	
Birth interval	Continuous	The time interval between consecutive births
Sex of child	0. Male 1. Female	
Size of the neonate at birth	0. Small 1. Average and above	mother’s estimate of the baby’s size at birth
Preceding birth interval	Continuous	The interval between the current birth and preceding births
Place of delivery	0. Home 1. Health facility	Health facility includes all private and public health facilities, including hospitals, health centres, clinics, health posts.
4+ antenatal care visits	0. No 1. Yes	
Initiated breastfeeding within an hour of birth	0. No 1. Yes	
Postnatal care use within two days of birth	0. No 1. Yes	

### Sampling

The samples for the EDHS survey were selected using a two-stage stratified cluster sampling technique using census enumeration areas (EA) as primary and households as secondary sampling units. An EA is a small geographic region with an average of 131 households [22]. The EDHS samples were stratified into urban and rural areas, and samples of EAs were selected independently in each stratum in two stages. The DHS sampling frame contains information about the EA location, type of residence (urban or rural), and the estimated number of residential households. In the selected EAs, a complete listing of households was carried out for each survey. Global Positioning System (GPS) data were collected at the level of each EA. Full detail of the methodology found in the main reports [24–26].

### Analysis

Descriptive data were summarized using the mean with standard deviation, median with first and third quartiles and percentages where appropriate. Spatiotemporal analysis was performed in two approaches: spatial scan statistics method and hotspot analysis methods. SaTScan™ software [27] using the Kulldorf’s method with Poisson purely spatial model was used to identify neonatal mortality cluster windows with associated mortality risks inside the cluster windows compared to surrounding areas. This approach produces trends of circular windows for areas with clusters of high neonatal mortality rates at each survey. The hot spot analysis performed in three steps. 1) Spatial autocorrelation statistics using the global Moran’s I index was estimated to explore the presence of clustering in the data. This procedure identifies the presence of positive or negative spatial autocorrelations. Positive spatial autocorrelation is the tendency for areas that are close to one another to have similar values of the variable (i.e., both high and both low counts of neonatal death). Negative spatial autocorrelation is the tendency for adjacent values to be dissimilar (i.e., high counts next to low counts of values) [28]. The value of spatial autocorrelation ranges from—1 to +1, where -1 is perfect clustering of dissimilar values; 0 is no autocorrelation, i.e., perfect randomness, and +1 indicates perfect clustering of similar values [29].

2) Once the presence of overall clustering is detected in the data, Getis-Ord Gi* statistics was used to identify the local hot spot areas. A hot spot area is an area with a statistically significant concentration of attributes (neonatal deaths) compared to its surrounding areas [30].

3) Spatiotemporal interpolation performed. Spatial interpolation is the process of using points with known values to estimate values at other points and is a means of creating surface data from sample points [31]. It is the process of obtaining a value for a variable of interest at a location where data has not been collected, using observed data [32, 33]. In this study, the Empirical Bayesian kriging (EBK) [34] method was used to interpolate data to the un-sampled areas.

Three-level logistic regression accounting for the nesting of household into clusters and clusters within regions was used to identify factors associated with neonatal mortality. Variables for the final model were screened using the Least Absolute Shrinkage and Selection Operator (Lesso) method. We implemented sample weighting in accordance with the recommendations provided in the DHS. Both standard and multilevel logistic regression models were fit and compared their performance using AIC, BIC, and deviance criteria. The comparison revealed that the multilevel model provided the best fit to the data. The Intra-class Correlation Coefficient (ICC) was calculated to assess the variation between clusters within regions, as well as the variation between regions. Adjusted Odds Rios (AOR) with corresponding 95% CI were presented as a measure of association and a P-value of 0.05 was used to declare statistical significance. ArcMap from ESRI ArcGIS Version 10.3 was used to perform the spatial analysis and R software version 4.2.2. was used for data manipulation and analysis [35].

### Ethical considerations

Formal approval to access and use the DHS dataset was obtained from the Demographic and Health Surveys Program. In this case, informed consent was not required because the analysis utilised secondary data and there was no direct contact with research participants.

## Results

### Socio-demographic characteristics of study participants

The study included 48,678 neonates, with the majority (88.0%) of participants living in rural settings. Over two-third (72.1%, 95% CI: 71.6, 72.6) of the mothers did not enrol in formal education, and their mean (SD) age when they gave their first birth was 18.4 (3.9). Over eight in ten births took place at home and less than one in ten births got postnatal care within two days of birth (Table 2). The median (Q1, Q3) birth size of babies was 3.1 (2, 4.3) kilograms. The median (Q1, Q3) number of children ever born was 4 (2.2, 6.7) and median (Q1, Q3) birth interval stood at 32 (23, 44) months.

**Table 2:** Socio-demographic and health service use characteristics of study participants, EDHS 2000–2019.

Variable	Frequency	Percentage (95% CI)	
		Unweighted	Weighted
**Marital status**			
Living together	45257	93 (92.7, 93.2)	93.5 (93.3, 93.7)
Not living together	3421	7 (6.8, 7.3)	6.5 (5.7, 7.3)
**Educational status of the mother**			
No education	34421	70.7 (70.3, 71.1)	72.1 (71.6, 72.6)
Primary	10335	21.2 (20.9, 21.6)	22.3 (21.5, 23.1)
Secondary and above	3922	8.1 (7.8, 8.3)	5.6 (4.9, 6.3)
**Place of residence**			
Urban	8338	17.1 (16.8, 17.5)	12 (11.3, 12.7)
Rural	40340	82.9 (82.5, 83.2)	88 (87.7, 88.3)
**Sex of child**			
Male	24943	51.2 (50.8, 51.7)	51.6 (51.0, 52.2)
Female	23735	48.8 (48.3, 49.2)	48.4 (47.8, 49.0)
**Received 4+ antenatal care**			
Yes	8178	16.8 (16.5, 17.1)	14.1 (13.3, 14.9)
No	40500	83.2 (82.9, 83.5)	85.9 (85.6, 86.2)
**Place of delivery**			
Health facility	9592	19.7 (19.4, 20.1)	15.1 (14.4, 15.8)
Home	39086	80.3(79.9, 80.6)	84.9 (84.5, 85.3)
**Received skilled birth attendance**			
Yes	11207	23 (22.6, 23.4)	19 (18.3, 19.7)
No	37471	77 (76.6, 77.4)	74.4 (74.0, 74.8)
**Initiated breastfeeding within an hour**			
Yes	21761	44.7 (44.3, 45.1)	45.1 (44.4, 45.8)
No	26917	55.3 (54.9, 55.7)	54.9 (54.3, 55.5)
**Received postnatal care within two days of birth**			
Yes	4461	9.2 (8.9, 9.4)	6.7 (6.0, 7.4)
No	44217	90.8 (90.6, 91.1)	93.3 (93.1, 93.5)

### Neonatal mortality rates

**Fig 1:**
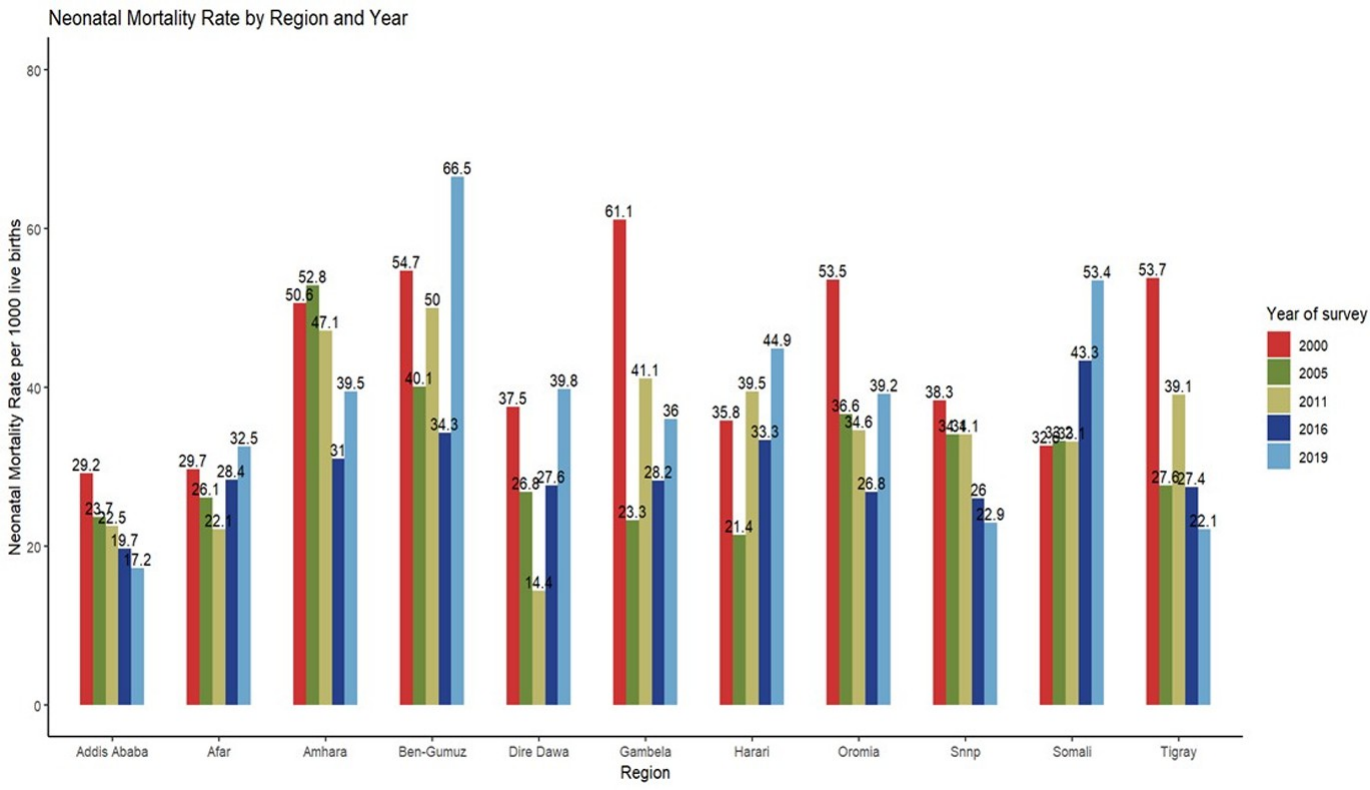
Neonatal mortality rates per 1000 live births by region and survey year (2000–2019). *SNNP = South nations nationalities and people region*.

In the 2000 survey, Gambella regional state had the highest neonatal mortality rates, followed by Benshangul Gumuz, Oromia, and Tigray regional states. In the 2005 survey, Amhara region took the lead in neonatal mortality, followed by Benshangul Gumuz and Oromia regional states. In the most recent 2019 survey, Benshangul Gumuz and Somali regional states sustained the highest neonatal mortality rates (Fig 1).

A significant disparity was observed in the average neonatal mortality rates across surveys between rural and urban areas, with rural areas experiencing an average neonatal mortality rate of 38 deaths per 1,000 live births while, urban areas had a comparatively lower rate of 29 deaths per 1,000 live births (p < 0.001). The percentage of neonatal mortality by demographic characteristics is presented in the supplementary file (S1 Table).

### Spatiotemporal patterns of neonatal mortality

**Fig 2:**
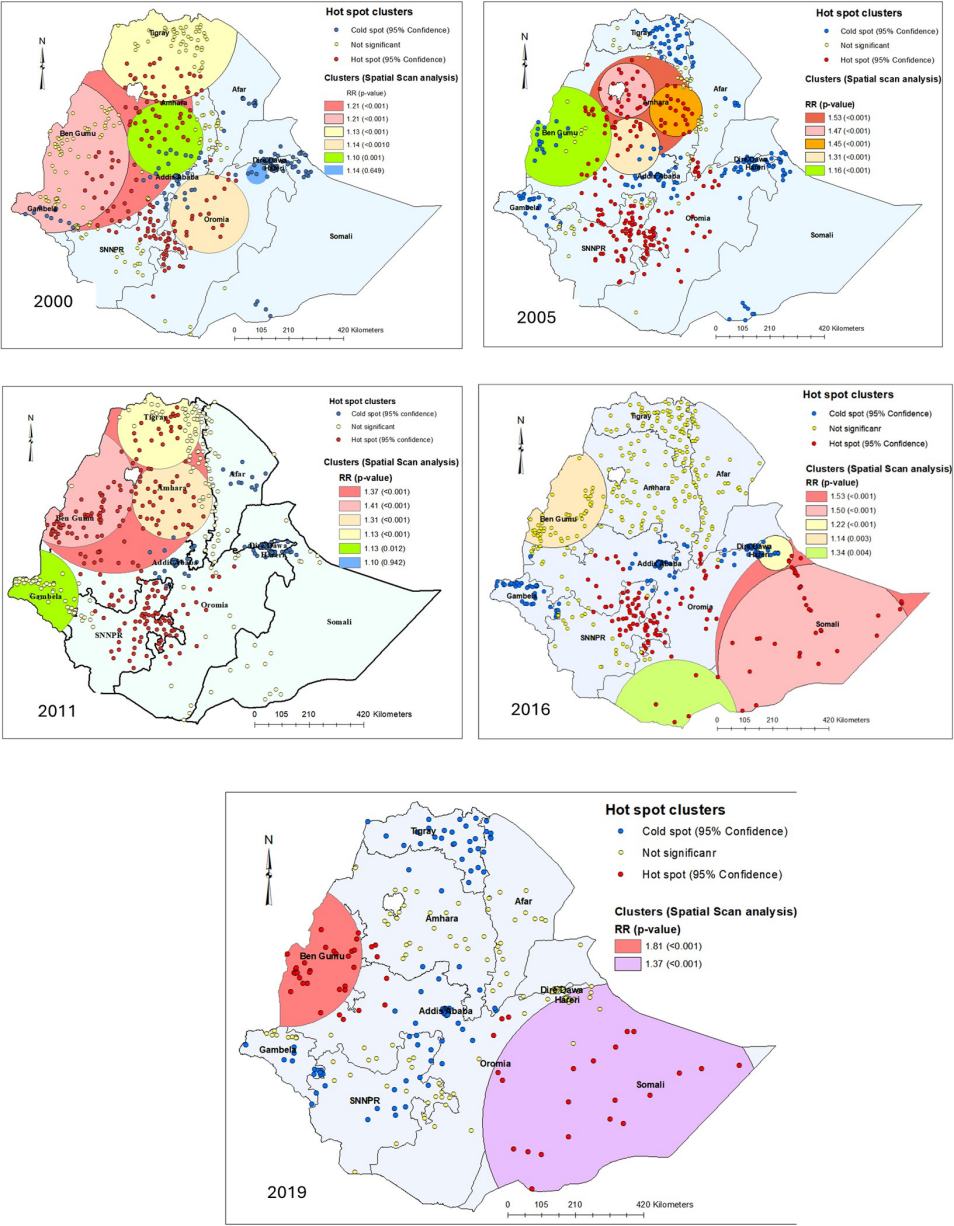
Spatiotemporal distributions of neonatal mortality in Ethiopia using DHS data between 2000 to 2019. The circles are significant clusters of neonatal mortality detected in SaTScan, and the red and blue dots are hot spots and cold spots identified in GIS, respectively (*Map source*: Natural Earth [36]). *Note*: *The relative risk (RR) with corresponding p-value of <0*.*05 indicated that the risk of neonatal mortality within the spatial window was higher compared to those outside the spatial window*.

In the 2000 survey, statistically significant clusters of neonatal mortality were identified in central west, southwest, northern, and north-western Ethiopia. Most of these clusters were found in the Amhara region, parts of Tigray, Oromia, and the former South Nations Nationalities and People Region (SNNPR). SaTScan analysis identified five significant cluster windows of neonatal mortality, with the majority of these clusters overlapping with the hotspot clusters identified by the Getis-Ord Gi* statistic (Fig 2).

The 2005 survey showed a pattern of neonatal mortality clusters that were roughly similar to those observed in the 2000 pattern, encompassing specific areas like parts of Oromia, the former SNNPR, and the entire Amhara regional state. Almost all parts of Amhara and Beneshangul Gumuz regional states and parts of Western Oromia were mapped with five clusters windows of neonatal mortality. In the 2011 survey, the hot spot clusters had expanded to cover a wider segment of Ethiopia, including the entirety of the Benshangul Gumuz, Amhara, and Tigray regions, as well as the majority of former SNNPR and Oromia regions.

**Fig 3:**
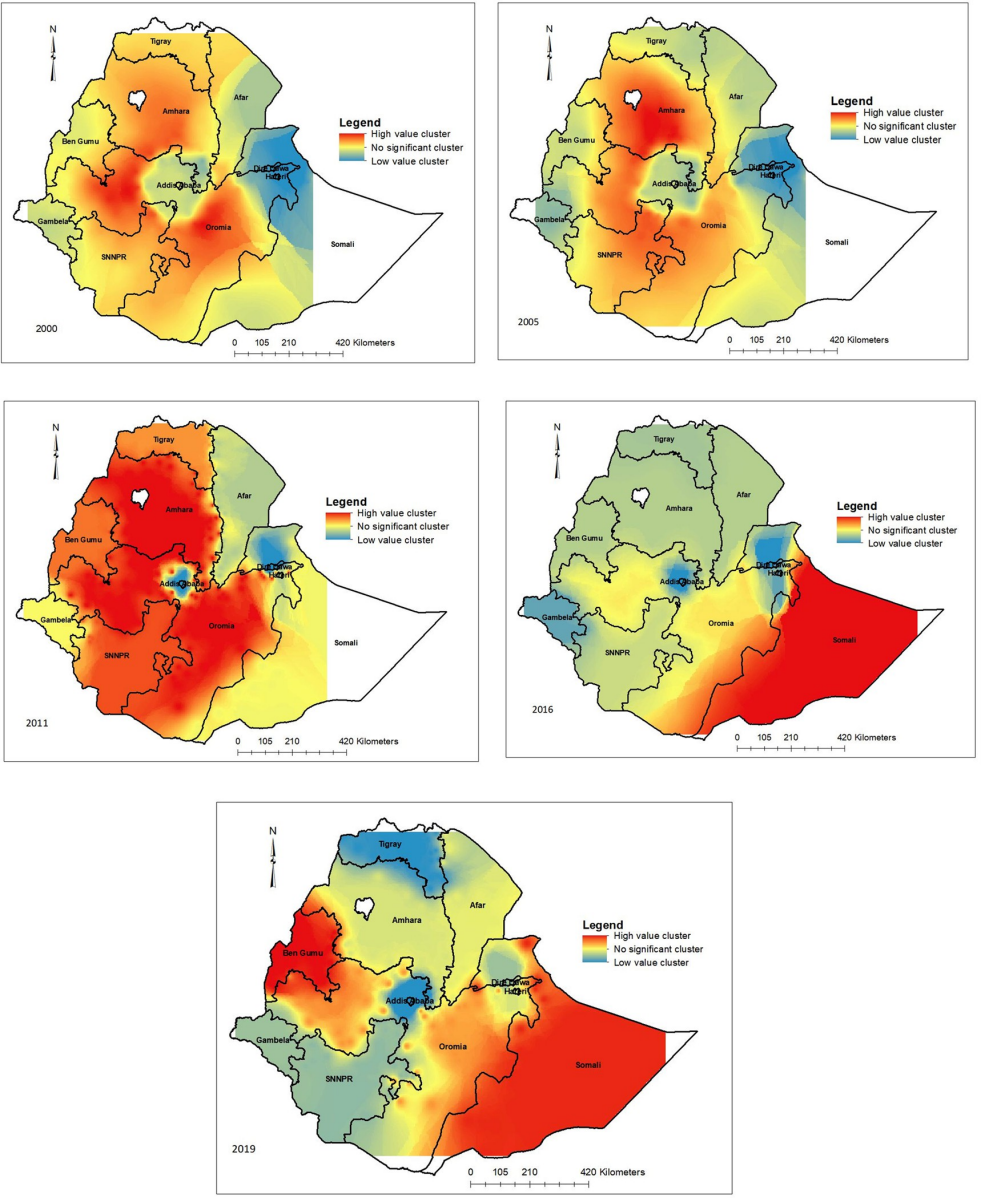
Spatial interpolated trends of neonatal mortality in Ethiopia from 2000–2019. The dark red colour indicates the hot spot clusters; the dark blue colour indicates the cold spot clusters, and the yellow ramp colour indicated areas with no significant clustering. *Note*: *White areas indicate no data available* (*Map source*: Natural Earth [36]).

In the two most recent surveys (2016 and 2019), most of the hot spot clusters of neonatal mortality were observed in the Eastern and South-Eastern parts of Ethiopia. However, spatial scan statistics identified cluster windows that encircle Western Ethiopia in both surveys as well as the Eastern and South-Eastern parts of the country. Statistically significant clusters in the 2016 survey were identified throughout the entire Somali region and in limited parts of South-eastern Oromia. In the 2019 survey, these clusters had expanded to the entire Somali region parts of eastern and central Oromia, the entirety of the Benshangul Gumuz region as well as adjacent areas in both the Oromia and Amhara regional states (Figs 2 and 3).

### Factors associated with neonatal mortality

The odds of neonatal mortality increased with the number of children a mother ever had (AOR = 1.36; 95% CI: 1.24, 1.51). Neonates born to mothers who did not live with a partner at the time of survey had a greater likelihood of death (AOR = 1.60; 95% CI: 1.26, 2.03) compared to those born to mothers living with a partner. The risk of neonatal mortality was higher among twin pregnancies compared to singletons (AOR = 5.02; 95% CI: 4.13, 6.10) and neonates delivered at home had a 46% increased risk of mortality compared to those born in health facilities (AOR = 1.46; 95% CI: 1.16, 1.82). Neonatal mortality was influenced by birth order (AOR = 0.99; 95% CI: 0.98, 0.99) and birth interval (AOR = 0.76; 95% CI: 0.68, 0.83), with higher birth order and longer time intervals between births showing a reduced neonatal death risk. Initiating breastfeeding within the first hour of birth was associated with reduced chances of neonatal death (AOR = 0.27; 95% CI: 0.23, 0.32). Male sex (AOR = 1.45; 95% CI: 1.28, 1.64) and those of smaller birth size babies (AOR = 1.08; 95% CI: 1.03, 1.12) were more likely to experience neonatal mortality compared to their counterparts (Table 3).

**Table 3:** Factors associated with neonatal mortality in Ethiopia, data from EDHS 2000–2019.

Variable	Category	Adjusted Odds Ratio (95% Cl)	P
Living situation of the mother	Living with partner	1	
	Not living with a partner	1.60 (1.26, 2.03)	<0.001
Place of residence	Urban	1	
	Rural	1.11 (0.88, 1.39)	0.390
Mother’s education status	No formal education	1	
	Primary education	1.01 (0.85, 1.19)	0.908
	Secondary and above	0.91 (0.63, 1.31)	0.601
Age at first birth		0.99 (0.97, 1.01)	0.210
Total children ever born		1.36 (1.24, 1.51)	<0.001
Four or more ANC visits	No	1	
	Yes	0.81 (0.63, 1.04)	0.101
Place of delivery	At home	1.46 (1.16, 1.82)	0.001
	Health facility	1	
Birth type	Singleton	1	
	Twin	5.02 (4.13, 6.10)	<0.001
Birth interval		0.99 (0.98, 0.99)	<0.001
Birth order		0.76 (0.68, 0.83)	<0.001
Initiated breastfeeding within an hour of birth	No		
	Yes	0.27 (0.23, 0.32)	<0.001
Postnatal care use within two days of birth	No	1	
	Yes	1.37 (0.99, 1.89)	0.054
Sex of child	Male	1.45 (1.28, 1.64)	<0.001
	Female	1	
Size of child at birth	Small	1.08 (1.03, 1.12)	0.002
	Average and above	1	
Model selection parameters	Fixed effects model	Random effects model	P -value
AIC	9241.0	9192.4	<0.001
BIC	9376.1	9344.4	
Deviance	9209.0	9156.4	
ICC cluster level		0.090	
ICC region level		0.176	

In the final model, approximately 9% of the variability in the outcome variable (neonatal mortality) was attributed to differences between regions, while a larger proportion, 17.6%, was attributed to differences between clusters, as indicated by the ICC values.

## Discussion

This study analyzed data from EDHS to explore the spatiotemporal patterns and identify contributing factors to neonatal mortality in Ethiopia from the year 2000 to 2019. The spatial analysis results identified areas with high neonatal mortality clusters. In the initial three surveys (2000, 2005 and 2016), certain areas were regularly identified as hot spot clusters. Specifically, the northern, north-western, and some central parts of Ethiopia were consistently associated with high rates of neonatal mortality. Regionally, the Amhara regional state was repeatedly pinpointed with hot spot clusters of neonatal mortality in the first three surveys. This is likely due to its limited access to health services and related infrastructure. For example, the Ministry of Health report based on the 2007 census indicated that the coverage of hospitals to population ratio in Amhara regional state was the lowest of any regional state in Ethiopia [37]. Another evidence from the 2008 Ethiopian emergency obstetric and neonatal care survey also showed that the percentage of the population served by a facility within a two-hour transfer time to obstetric emergency care service in Amhara region was 68% compared to the nearby Tigray region which was 80% [38]. The 2014 Service Provision and Availability (SPA) survey report also indicated that the Amhara region was among the lowest in health facilities infrastructure based on the basic amenities domain for assessing general service readiness within the health facility assessment methodology proposed by WHO and USAID. For example, the regular electricity, connection to a power grid, improved water source and piped water sources availability were 45%, 65%, 79% and 50% compared to the 64%, 83%, 86% and 69% coverage in the nearby Tigray regional state respectively [39].

The Beshangul Gumuz and Somali regional states were also consistently mapped with hot spot clusters in two out of the last three surveys. The health service coverage in these regions was also relatively lower. For example there were only two hospitals at a regional level in Beshangul Gumuz in 2007 [37] and the regular electricity, connection to a power grid, improved water source and piped water sources availability were at a lower coverage in the region at 47%, 70%, 76% and 36% respectively [39]. The Somali region was also among the lowest in health facilities infrastructure [39].

The study also identified factors associated with neonatal mortality. Neonates born at home were 46% more likely to die compared to their counterparts delivered in health facilities. This can be attributed to the absence of medical amenities at home, which are crucial for ensuring safe births and managing various birth complications, such as asphyxia, infections, and preterm births, which are common causes of neonatal death. This adversely impacts the survival chances of newborns. Our finding is in line with studies from developing countries, which have found that neonatal mortality is higher among those born at home than those born in medical facilities [40, 41].

The living situation of mothers significantly influenced neonatal mortality, with those not living with a partner having higher odds of neonatal mortality. This can be attributed to the fact that mothers who do not live with a partner often face greater socioeconomic and psychological challenges, which can directly impact the health and survival of their neonates. Initiating breastfeeding within the first hour of birth was associated with reduced chances of neonatal death. This is due to the fact that the mother’s first milk after childbirth, known as colostrum, supplies essential antibodies and vital nutrients. It essentially serves as an initial immunisation for the newborn, bolstering their immune system and decreasing the likelihood of mortality during the neonatal stage [42].

Having smaller birth weight was associated with increased neonatal mortality. In line with other literature low birth weight, poses a significant public health concern, as it correlates with a higher mortality rate among neonates [43, 44].

The likelihood of neonatal mortality was greater for twin pregnancies than for single births. This is because multiple pregnancies elevate the chances of complications during pregnancy and childbirth, including a heightened potential for birth anomalies and infections [45, 46]. Twin pregnancies present additional hazards for newborns, primarily because of premature labour, which often leads to low birth weight–a primary factor influencing neonatal mortality [47, 48].

Our results highlight the importance of improving access to quality antenatal and delivery care, promoting essential newborn care practices, enhancing infection control, empowering communities through education, and strengthening healthcare infrastructure.

### Strengths and limitations of the study

This study used nationally representative data from all available DHS surveys in Ethiopia. However, as the analysis was based on secondary data, it would have faced the inherent limitations of the DHS surveys, including reporting and recall biases associated with retrospective data that depends on recollections of past events as well as lack of data on potential contributors to neonatal mortality such as clinical factors. Additionally, there was geographical displacement of DHS survey GPS locations for privacy reasons that may affect the interpretation of results. However, we have mitigated potential misinterpretations by focusing on larger area-level interpretations.

## Conclusions

Neonates born at home, those who did not start breastfeeding within the first hour after birth, males, and those with smaller birth sizes were found to have a higher likelihood of neonatal mortality. In the first three surveys, most of the significant clusters were predominantly found in the central southern, central-western, north-western, and northern parts of Ethiopia. However, in the two most recent surveys, the clustering shifted towards the Eastern and South-eastern areas of the country. Policymakers and resource administrators at different levels should give special emphasis to areas identified with significant clusters of neonatal mortality. The Ethiopian government should ensure that essential neonatal services and supplies are available in healthcare facilities, especially in rural and remote areas.

## Supporting information

S1 Table Percentage summary of demographics by outcome variable. (DOCX)

## List of abbreviations

AOR: Adjusted Odds Ratio
CI: Confidence Interval
EA: Enumeration area
EDHS: Ethiopian Demographic and Health Surveys
GIS: Geographic Information System
SNNP: South nations nationalities and people region
SPA: Service Provision and Availability
USAID: United States Agency for International Development
WHO: World Health Organization
