# Supplementary material for: Spatiotemporal patterns and factors contributing to neonatal mortality in Ethiopia: Data from EDHS 2000 to 2019
Source: PLoS One. 2024 Nov 4;19(11):e0310276. doi: 10.1371/journal.pone.0310276 (PMC11534208; doi:10.1371/journal.pone.0310276)
Supplement: S1 Table — (DOCX) [file pone.0310276.s001.docx]

Table S1: Percentage summary of demographics by outcome variable.

| **Variable** | **Neonatal mortality** |  |  | **Yes** |
| --- | --- | --- | --- | --- |
|  | **No** |  |  |  |
| **Marital status** |  | | |  |
| Living together | 93.6 | | | 91.1 |
| Not living together | 6.4 | | | 8.9 |
| **Educational status of the mother** |  | | |  |
| No education | 72 | | | 75 |
| Primary | 22.4 | | | 20.9 |
| Secondary and above | 5.7 | | | 4.1 |
| **Place of residence** |  | | |  |
| Urban | 12 | | | 11.3 |
| Rural | 88 | | | 88.7 |
| **Sex of child** |  | | |  |
| Male | 51.2 | | | 61.7 |
| Female | 48.8 | | | 38.3 |
| **Received 4+ antenatal care** |  | | |  |
| Yes | 14.4 | | | 8.2 |
| No | 85.6 | | | 91.8 |
| **Place of delivery** |  | | |  |
| Health facility | 15.1 | | | 15.7 |
| Home | 84.9 | | | 84.3 |
| **Received skilled birth attendance** |  | | |  |
| Yes | 19.1 | | | 15.4 |
| No | 74.3 | | | 76.9 |
| **Initiated breastfeeding within an hour** |  | | |  |
| Yes | 46.3 | | | 14.5 |
| No | 53.7 | | | 85.5 |
| **Received postnatal care within two days of birth** |  | | |  |
| Yes | 6.7 | | | 6.7 |
| No | 93.3 | | | 93.3 |
